# Study on Static and Fatigue Behaviors of Steel-UHPFRC Composite Deck Structure

**DOI:** 10.3390/polym14142796

**Published:** 2022-07-08

**Authors:** Jun Luo, Chenzi Huai, Xudong Shao, Jun Zhao, Ling Wang

**Affiliations:** 1 School of Mechanics and Safety Engineering, Zhengzhou University, Zhengzhou 450001, China; luojun@zzu.edu.cn; 2 Yellow River Engineering Consulting Co., Ltd., Zhengzhou 450003, China; huaichenzi@126.com (C.H.); wangling@yrec.cn (L.W.); 3 School of Civil Engineering, Hunan University, Changsha 410082, China

**Keywords:** lightweight composite deck (LWCD), orthotropic steel deck (OSD), bending test, fatigue test, cracking stress

## Abstract

Ultra-high-performance fiber-reinforced cementitious composite (UHPFRC) is used in orthotropic steel deck (OSD) to form a lightweight composite deck structure (LWCD), which is expected to solve the problems of fatigue cracking of traditional steel deck and pavement damage. This paper aims to study the influence of key design parameters on longitudinal bending and transverse fatigue performance, as well as the ultimate bearing capacity calculation theory of the LWCD. A local finite-element (FE) model was built to evaluate the vehicle-induced stress ranges of six typical fatigue-prone details. In total, eight negative bending tests on steel-UHPFRC composite beams and one fatigue test on a steel-UHPFRC composite plate were conducted to investigate the longitudinal bending performance and the transverse flexural fatigue behavior of the LWCD, respectively. The results show that adding a 60-mm UHPFRC layer can significantly reduce the stress amplitude of six typical fatigue details by 44.8% to 90%. The failure mode of the longitudinal bending tests is the U-rib buckle and all UHPFRC layers exhibit multiple cracking behaviors when the specimens failed. The longitudinal cracking stresses of the specimens are between 20.0 MPa to 27.3 MPa. The reinforcement ratio and cover thickness have a great influence on the cracking stress. While the ultimate bearing capacity of specimens with different parameters has little difference. The calculation method of the ultimate bearing capacity of a steel-UHPFRC composite structure is proposed. When the strain at the bottom of the u-rib is taken as 1.2 times the design yield strain, the calculated results are in good agreement with the experimental results. No fatigue failure was observed after 66.12 million fatigue cycles under the design load, highlighting the favorable fatigue resistance of the proposed LWCD.

## 1. Introduction

Orthotropic steel decks (OSD) have been widely used for the construction of long-span steel bridges because of their high longitudinal stiffness, relatively small self-weight, and excellent seismic-resisting performance 1. While fatigue cracking in a conventional OSD system and damage to asphalt overlay are frequently reported all over the world because of poor fatigue resistance of welded details of OSD, large heavy traffic volumes, and serious overload [1,2]. In order to enhance the low fatigue resistance of OSD, a lightweight composite deck system (LWCD) using ultra-high performance fiber reinforced cementitious composite (UHPFRC) has been developed by Shao et al. [3]. In the LWCD, a UHPFRC layer (normally 35–60 mm in thickness and containing steel reinforcement bars) is added on top of the OSD and connected by shear studs. Thus, the stiffness of composite deck structure is significantly improved and vehicle-induced stress range in critical welded details of OSD decreases extending the fatigue life.

In recent years, some experiments have been performed to investigate the bending behaviors or fatigue behaviors of UHPC members [4,5] and UHPC-NC composite beams [6,7,8,9]. The results indicated that the UHPC layer can improve the structural performance significantly regarding ultimate loads, stiffness, and cracking behaviors. W. Lorenc [9] carried out extensive research into the failure mechanisms and positive bending behavior of composite steel–concrete beams prestressed with external tendons. It was found that the tendon shape has no significant effect on the behavior and ultimate resistance of composite steel–concrete beams at the same eccentricity of tendons. It is also shown that steel–concrete bond cohesion can significantly influence the behavior of the shear connection in composite beams.

In addition, some scholars have conducted some research on the mechanical performance of steel-UHPFRC composite structures. Peter Buitelaar [10] showed that the rehabilitation and strengthening of OSD with reinforced high-performance concrete overlay can extend the service life of OSD by improving the stiffness. Dieng et al. [11] and Choi [12] found that adding a thin UHPFRC layer to OSD could reduce the stresses of this composite structure and the thickness of UHPFRC affected the appearance and propagation of cracks. Furthermore, the research group at Hunan University conducted a series of experimental studies on the LWCD. Shao et al. [3,13,14] investigated the longitudinal bending performance of steel-UHPFRC composite beam with 45–50 mm UHPFRC and small cover thickness. It revealed that the steel-UHPFRC composite beams with 15-mm cover thickness have high cracking strength and the cover thickness has a large influence on the cracking strength. Luo et al. [15] studied the influence of main design parameters (UHPC thickness, cover thickness, stud spacing, and reinforcement ratio) on the transverse bending performance of composite deck structure through 40 bending tests of steel-UHPC composite plates, the results showed that the reinforcement ratio and the cover thickness have a great influence on the cracking stress. When the cover thickness is large or the reinforcement ratio is reduced, the cracking stress is significantly reduced. Zhang et al. [16,17,18,19] studied the fatigue performance in the longitudinal direction of LWCD experimentally and theoretically. The results showed that the use of a thin UHPFRC (45–50 mm) layer could decrease the fatigue stress in OSD by 30–80% and the LWCD has favorable fatigue performances in the longitudinal direction. M. Kożuch and W. Lorenc [20,21,22] studied the elastic resistance, calculation method of stress, and design concept of the shear connection. The results showed that It is possible to determine the state of stress in the connector for any combination of internal forces acting on the composite beam, based on the combination of results of two fragmentary FE models. In addition, the proposed simplified design method can cover many types of composite sections, externally reinforced sections, and conventional composite sections.

It can be seen from the aforementioned studies that the calculation method of stress and design concept of the shear connection, the longitudinal bending fatigue performance, and the influence of main design parameters on the transverse bending performance of LWCD were studied and the cover thickness has a great influence on the cracking stress, while, the research on the transverse bending fatigue behavior and the influence of main design parameters on the longitudinal bending performance of LWCD is limited. On the other hand, it is known that the corrosion resistance of reinforcement bars is closely related to the cover thickness. When the thickness of the protective layer is small (e.g., ≤15 mm), the construction accuracy is high and difficult to be controlled in practice. Thus, an unexpected weakness may exist in the structure. In addition, UHPFRC material has good durability, such as better impermeability, chloride ion permeability, and wear resistance which is about three times that of ordinary concrete [23]. Thus, when the cover thickness is big (e.g., 25 mm), the LWCD can be used for real projects without overlay if the mechanical properties can meet the design requirements. Thus, it is of great significance to study the mechanical performance of LWCD with a large cover thickness. Therefore, this paper conducts a finite element analysis and a series of tests to study the transverse fatigue performance and longitudinal bending performance of the LWCD structure. The experimental campaign includes longitudinal bending tests with different design parameters and transverse fatigue tests with 60-mm UHPFRC layer thickness and 25-mm cover thickness.

## 2. Finite Element Analysis (FEA) of the LWCD

### 2.1. Basic Information of Humen Bridge

This paper takes the Humen Bridge, a steel box girder suspension bridge with a main span of 888 m, as the engineering background. The Humen Bridge was opened to traffic in 1997 and its rise-to-span ratio is from 1 to 10.5. The width and structural depth of the steel girder are 35.6 m and 3.012 m, respectively. The thickness of the orthotropic deck plate bridge is 12 mm, and the longitudinal stiffener is a U-shaped stiffener with a thickness of 8 mm. The trough ribs are 260 mm high and placed at 620-mm centers. The top width and bottom width of the U-rib are 320 mm and 210 mm, respectively. The distance between the adjacent transverse diaphragm is 4 m [24].

**Figure 1:**
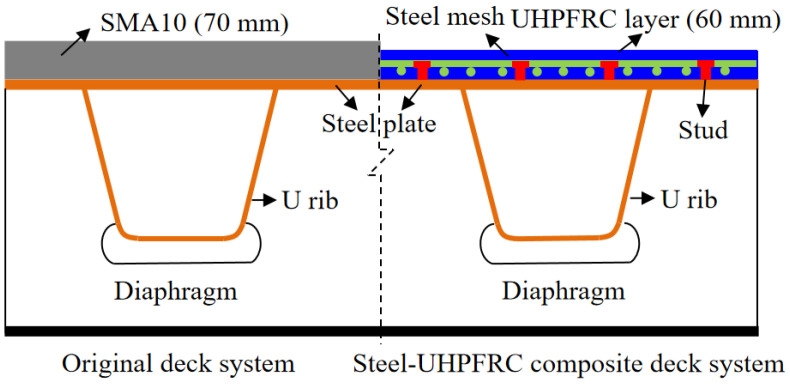
Two different bridge deck systems.

The LWCD is proposed to be applied on the bridge. The specific design scheme is as follows: the original pavement layer is removed firstly, then the short-headed stud with a length of 35 mm and diameter of 13 mm is welded on the original orthotropic deck plate. Afterward, the reinforcement bars in both longitudinal and transverse directions are arranged, and the UHPFRC layer with a thickness of 60 mm is poured on-site. The original and proposed composite deck systems are shown in Figure 1.

### 2.2. Local FE Model

**Figure 2:**
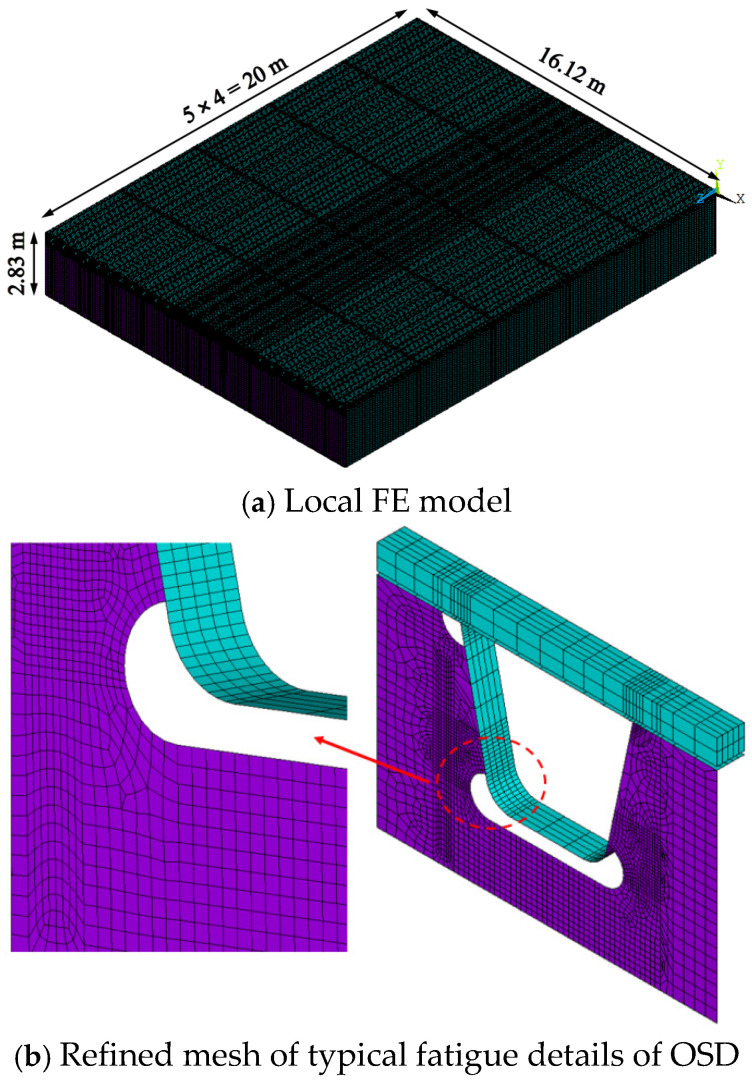
FE model of LWCD.

According to the modeling method in the reference [24], a local FE model of a steel box girder with a 60-mm UHPFRC layer is established using *ANSYS*. The model takes a length of five spans of the diaphragm in the longitudinal bridge direction. For simplification, only half-frame box beam structure in the transverse direction is simulated, and the secondary structures such as wind nozzles and manholes are not considered. The FE model is shown in Figure 2.

In the FE model, the top plate, bottom plate, U-rib, web, and diaphragm are all simulated by SHELL63. The UHPFRC layer is simulated by SOLID45. The sheer force of studs is considered by defining the transverse and longitudinal stiffness. The short-headed studs are simulated by CONBIN 14 and the shear stiffness is 120 kN/mm. The constitutive laws of the materials and the boundary conditions of the model are the same as those in the literature [24]. Because the tensile stress and compressive stress of the UHPFRC layer applied to the simulated bridge are much smaller than their compressive and tensile strengths, that is, the UHPFRC only utilizes a relatively small stress level. Therefore, the material property of UHPFRC is also assumed to be linear elasticity in the calculation. The load is applied based on the standard fatigue model 3 in the Specifications for Design of Highway Steel Bridge in China (JTG D64-2015) [25]. According to the geometrical characteristics of OSD, there are three kinds of loading modes in the transverse bridge direction, including above the U-shaped rib, riding the U-shaped rib, and between the U-shaped ribs. And two types of loading modes in the longitudinal bridge direction, namely the mid-span position and above the diaphragm. During the analysis, the stress state of UHPFRC and typical fatigue details of OSD are mainly concerned.

**Figure 3:**
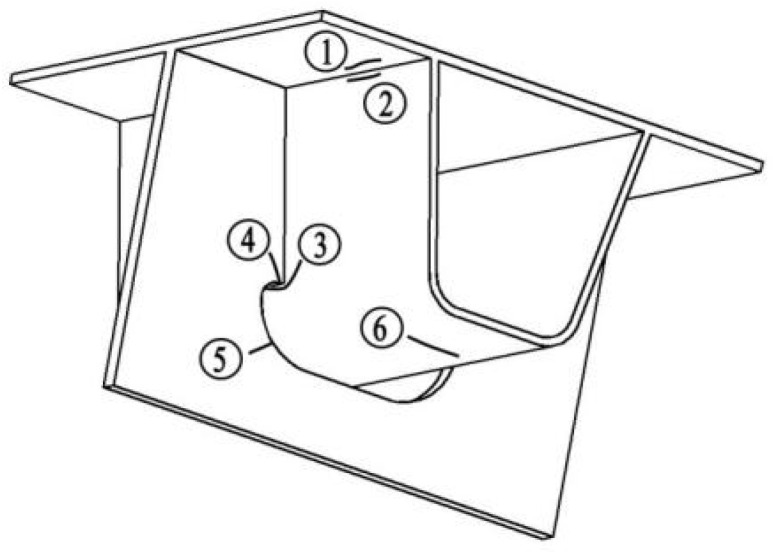
Typical fatigue details of steel bridge deck.

Pfeil [1], Zhan [24], Sim et al. [26] have conducted a series of researches on the typical fatigue cracking details of steel bridge decks. The results show that the details of the steel bridge deck prone to fatigue cracking are shown in Figure 3 [24]: ① longitudinal crack detail of steel plate at the weld area between steel plate and U-rib; ② longitudinal crack detail of U-rib at the weld area between steel plate and U-rib; ③ crack detail of web at the intersection area between U-rib and diaphragm; ④ crack detail of diaphragm at the intersection area between U-rib and diaphragm; ⑤ crack detail at the area of arc incision; ⑥ crack detail of butt weld area at the bottom of U-rib.

### 2.3. Main Analysis Results

The maximum tensile stress of UHPFRC in the transverse bridge direction is 3.8 MPa. As shown in Table 1, adding a 60-mm UHPFRC layer can significantly reduce the stress amplitude of six typical fatigue details mentioned above and the reduction degree ranges from 44.8% to 90%. Thus, the risk of fatigue cracking can be effectively reduced.

## 3. Experimental Methodology

### 3.1. Test Program

In order to investigate the transverse fatigue performance and the influence of main design parameters on the longitudinal bending performance of LWCD, a series of full-scale tests were carried out. According to the structural and stress characteristics of OSD, for clarity and design convenience, the longitudinal mechanical performance can be studied by a simplified stiffened steel plate deck consisting of the longitudinal ribs and the transverse mechanical behavior can be studied by a simplified deck plate acting between longitudinal ribs [15,27]. Accordingly, two different test series including one steel-UHPFRC composite plate and eight steel-UHPFRC composite beams were fabricated to investigate the transverse fatigue behavior and longitudinal bending performances of the steel-UHPFRC composite deck structure, respectively. The parameters of the components are shown in Table 2. All of them were used for the negative four-point bending test.

### 3.2. Materials

**Figure 4:**
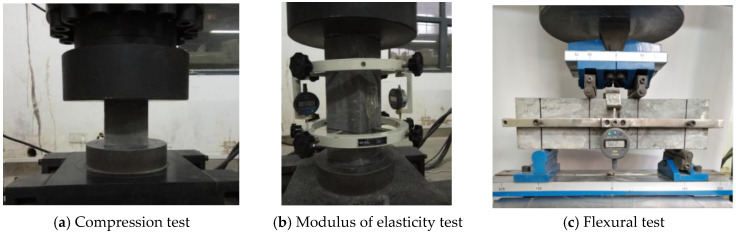
Test of material properties.

**Figure 5:**
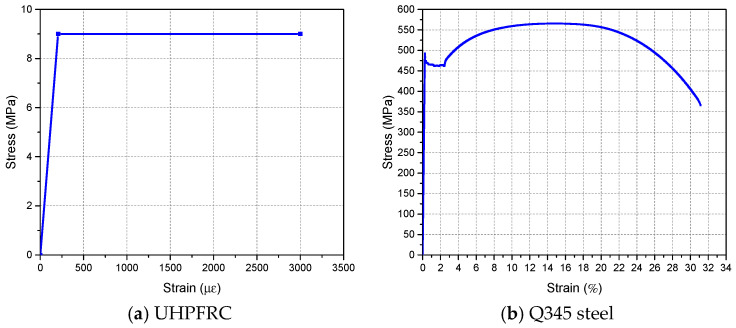

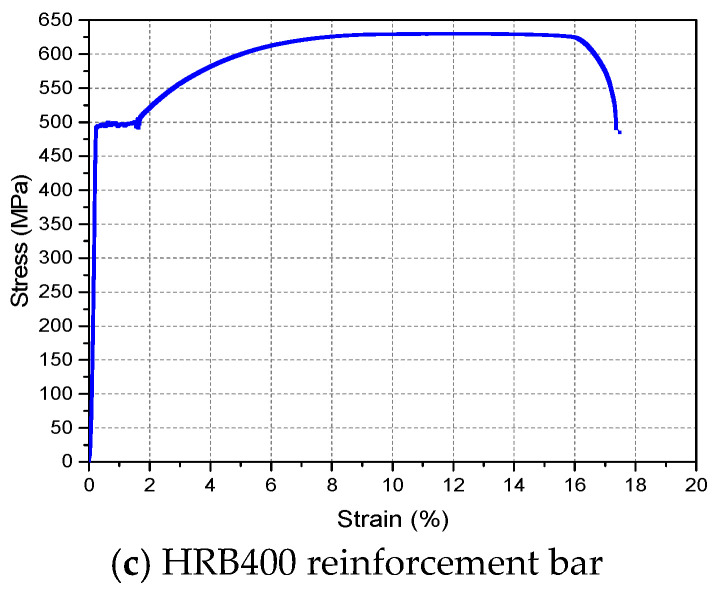
Stress-strain curves of materials in tension.

The UHPFRC used in this test was developed and produced by the UHPC R & D team of Hunan University, China. It is composed of cement, silica fume, quartz sand, steel fibers, quartz powder, water, etc. In order to obtain the mechanical properties of UHPFRC, standard UHPFRC specimens for mechanical properties characterization were fabricated and cured under the same conditions as the steel-UHPFRC composite specimens [28]. Relevant information and test results of mechanical property tests of UHPFRC material are shown in Table 3. The test photos are shown in Figure 4. The test results showed that the compressive strength, flexural strength, and modulus of elasticity of UHPFRC were 165.0 MPa, 30.1 MPa, and 45.8 GPa, respectively. Furthermore, based on the flexural strength of UHPFRC and the calculation method of tensile strength recommended by AFGC/SETRA [29], the calculated tensile strength of UHPFRC was 9.0 MPa and the simplified stress-strain curve of UHPFRC was shown in Figure 5a.

The steel plate used for making components is a Q345 steel plate and the nominal yield strength is 345 MPa. The grade of reinforcement bars is HRB400 and the diameter is 10 mm. The measured stress-strain curves in the tension of Q345 steel and HRB400 reinforcement bar were shown in Figure 5b,c. The UHPFRC layer and the steel plate are connected by short studs, its diameter is 13 mm, and the length after welding is 35 mm.

### 3.3. Main Fabrication Process of Steel-UHPFRC Composite Specimens

**Figure 6:**
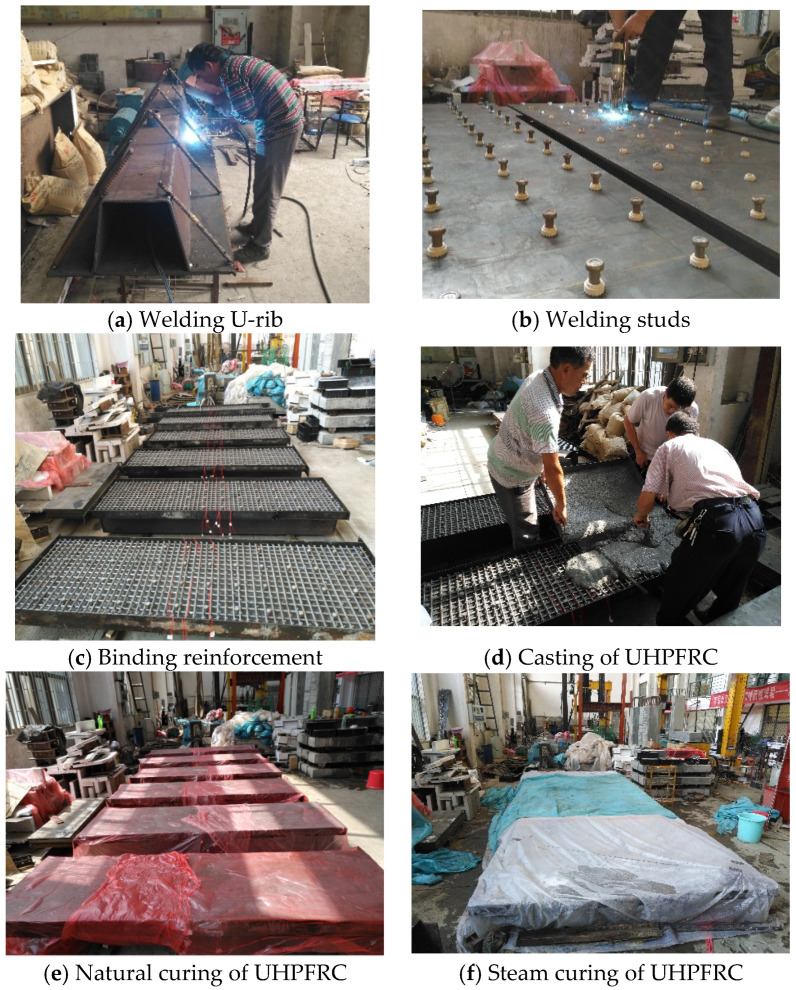
Main specimen preparation procedures.

The fabrication of steel-UHPFRC composite components mainly includes the following processes: (1) the U-rib was welded to the steel plate; (2) short studs were welded to the steel plates; (3) steel frameworks were supported and reinforcement mesh was bound; (4) UHPFRC was poured from one side; (5) Natural curing of UHPFRC for 48 h; (6) High-temperature steam curing (90–100 °C) of UHPFRC for 48 h. Figure 6 presents the main specimen preparation procedures.

## 4. Longitudinal Bending Tests of the LWCD

### 4.1. Design of Full-Scale Steel-UHPFRC Composite Beams

**Figure 7:**
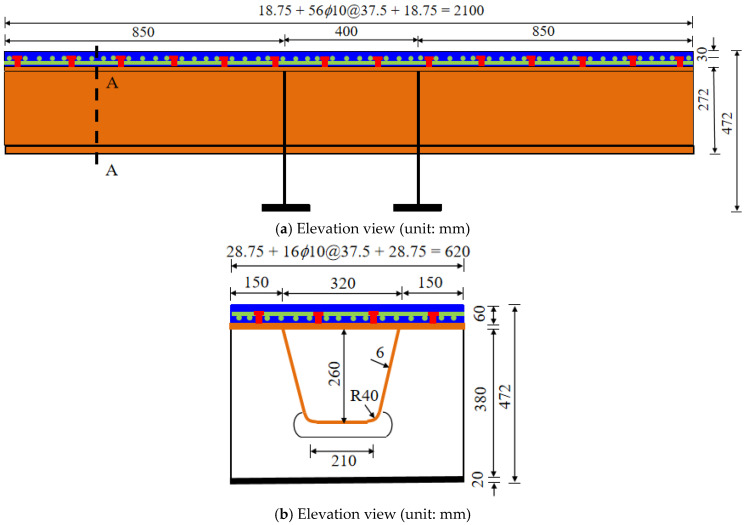
Details of steel-UHPFRC composite beam (take U155-60-25-16 as an example).

A total of eight full-scale strip models of steel-UHPFRC composite beams with different design parameters were designed for the test. The test includes three design variables: (1) UHPFRC layer thickness; (2) Cover thickness; (3) Number of longitudinal reinforcement. The spacing of studs is 155 mm, the thickness of the UHPFRC layer is 45 mm/60 mm, the cover thickness is 15 mm/25 mm, and the number of longitudinal reinforcement is 12/16. Taking component U155-60-25-16 as an example, the structural diagram of the component is shown in Figure 7. The structural diagrams of other components are similar to this, which are only different in the combination of main design parameters and will not be listed one by one. The total length of the specimen is 2100 mm, the width is 620 mm, the thickness of the steel deck is 12 mm, the thickness of the u-stiffener is 6 mm and the height is 260 mm. The spacing of longitudinal and transverse reinforcement mesh is 37.5 mm. In order to facilitate loading and prevent local yielding of U-rib, two transverse diaphragms with a spacing of 400 mm are set in the middle of the beam.

### 4.2. Loading Scheme and Test Contents

**Figure 8:**
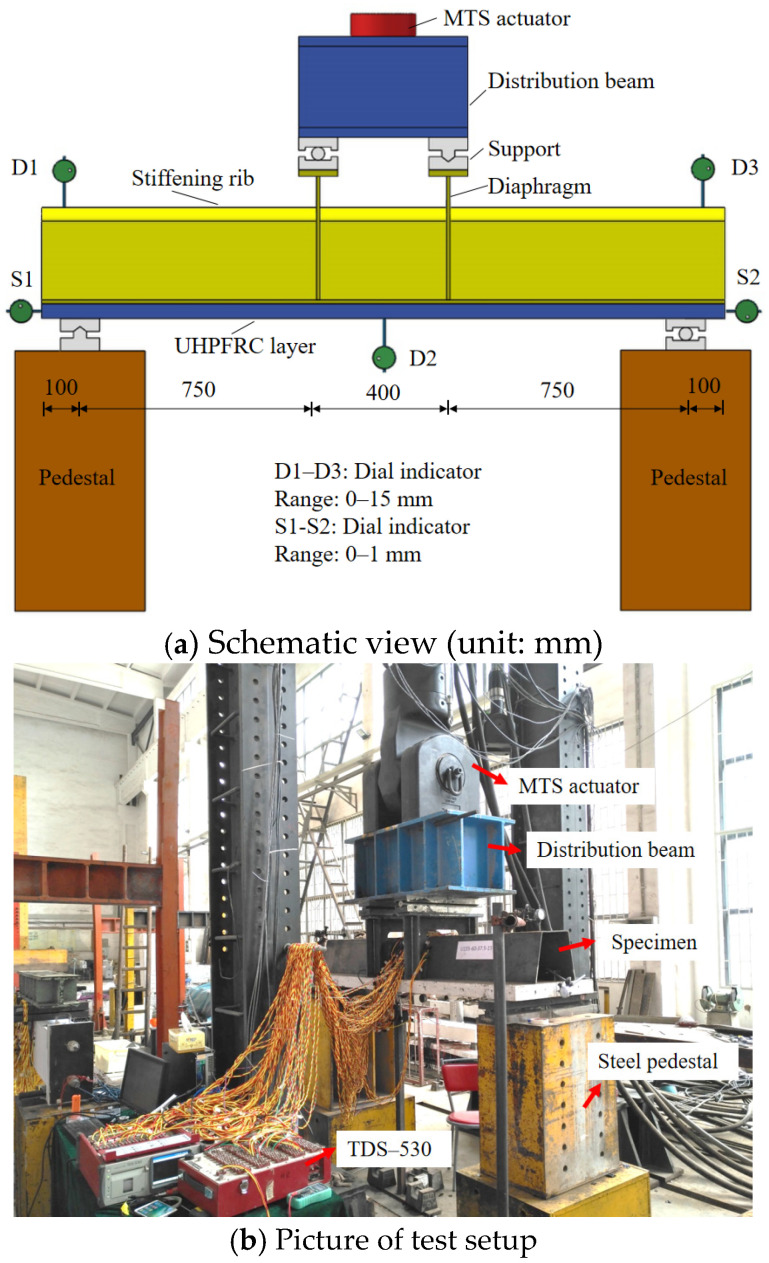
Test setup of steel-UHPFRC composite beam.

**Figure 9:**
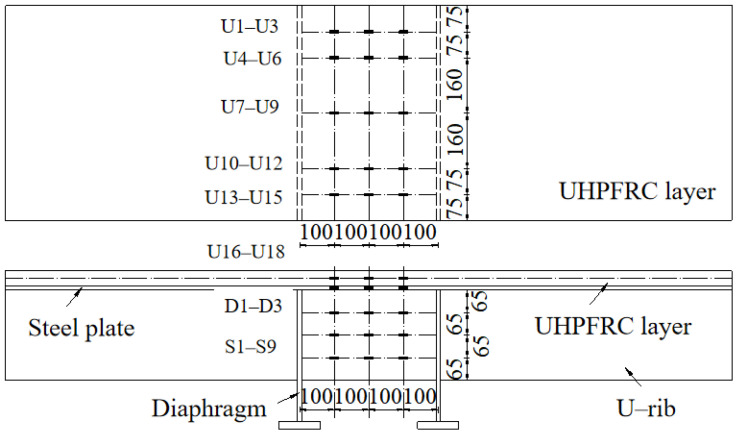
Layout of strain gauges (unit: mm).

Similarly, the four-point negative bending test method was applied on the steel-UHPFRC composite beams with a UHPFRC layer in tension, as shown in Figure 8. The hydraulically actuated testing machine MTS with a large capacity was used, and the load was applied on the two diaphragms with a spacing of 400 mm. Dial indicators D1, D2, and D3 were used to measure the mid-span displacement and the displacement at both ends of the support, respectively. Dial indicators S1 and S2 were used to measure the slip between the steel and UHPFRC layers. Along the longitudinal direction of the member, strain gauges were arranged at the quartering points of the pure bending section to measure the strain of UHPFRC, steel and reinforcement, as shown in Figure 9.

During testing, the force-based control with a loading range of 10 kN/level was applied in the elastic stage and switched to displacement-control after the elastic stage. The displacement, the local deformation of the U rib, the interface slip between the steel and UHPFRC layer, the strain data under each level of load, the cracking, crack width, and development on the top surface of UHPFRC within the pure bending section were mainly concerned.

### 4.3. Experimental Results and Discussion

#### 4.3.1. Force-Midspan Deflection Response

**Figure 10:**
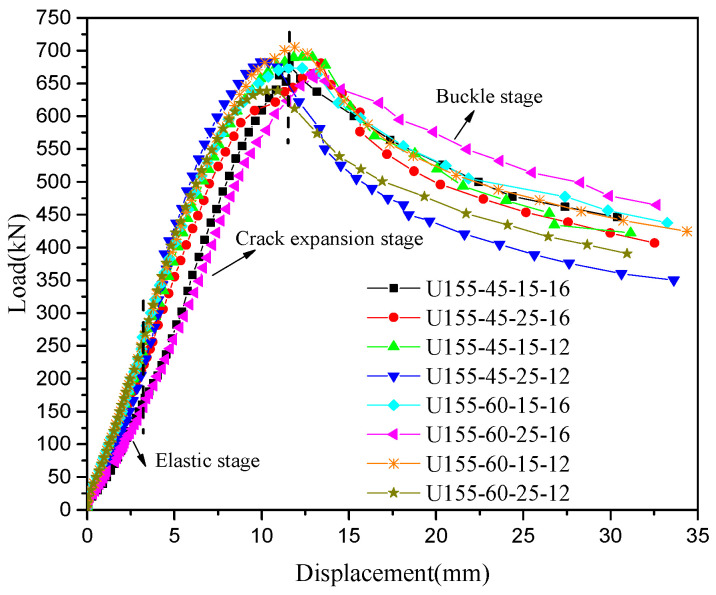
Load-deflection curves of steel-UHPFRC composite beams.

**Figure 11:**
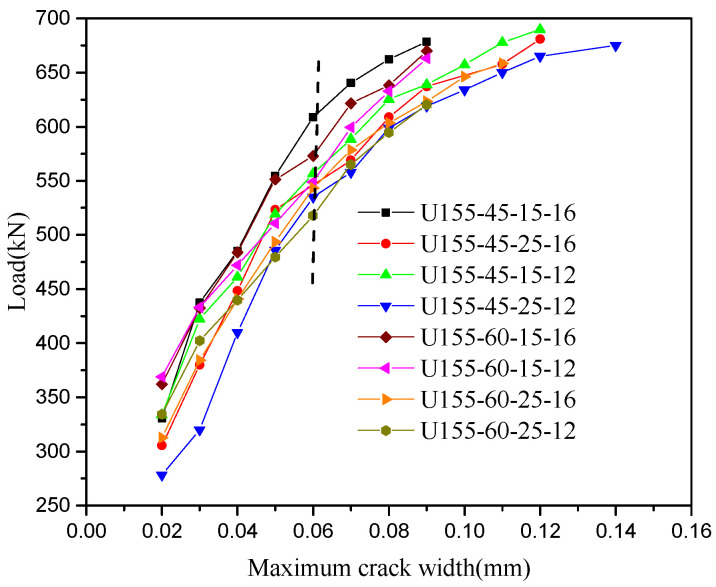
Load-maximum crack width curves of steel-UHPFRC composite beams.

**Figure 12:**
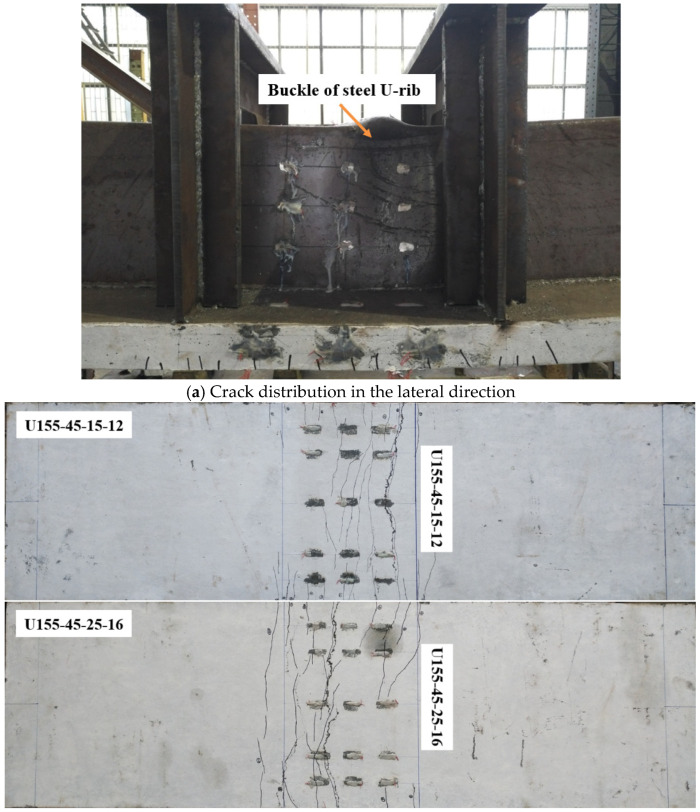

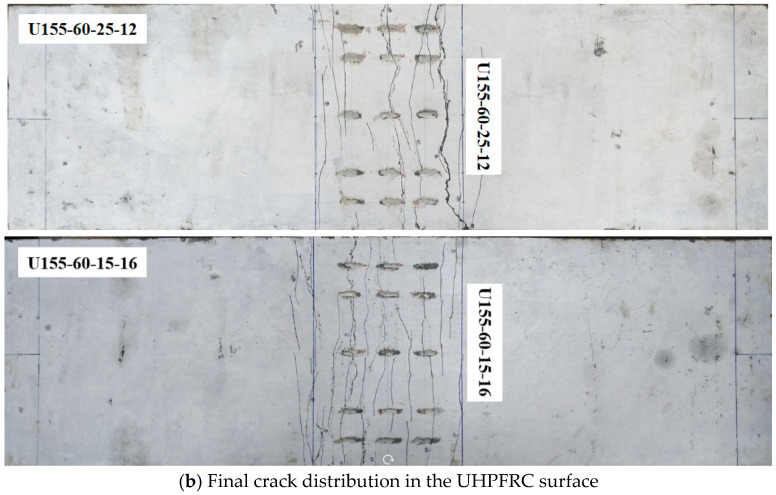
Final crack distribution of some components.

The load-midspan deflection curves of eight steel-UHPFRC composite beams are presented in Figure 10. As shown in Figure 10, the load-deflection curves include three different stages, namely, the elastic stage, crack expansion stage, and buckle stage. In the elastic stage, there is no crack, and the load midspan displacement curve is approximately a straight line, that is to say, both UHPFRC and steel U-rib exhibited elastic behavior. From the elastic stage to the yield of the U-rib of the composite beam is the crack propagation stage, and all the specimens fail due to the yield of the U-rib. In the crack propagation phase, for steel-UHPFRC composite beams, cracks appear on the surface of UHPFRC, and with the increase of load, the number of cracks increases, and the width of cracks gradually increases, as shown in Figure 11. While the stiffness is basically similar to that in the elastic stage and there is no obvious reduction. On the other hand, the load-midspan displacement curves and the ultimate bearing capacity of specimens with different parameters have little difference. When the bottom of the U-rib yields, as shown in Figure 12a, the member enters the yield stage. In the yield stage, the bearing capacity of the member decreases rapidly and the midspan displacement increases rapidly. Moreover, the crack width continues to increase. The final crack distribution of the steel-UHPFRC composite beam is shown in Figure 12b. It can be seen that the cracks on the surface and side of UHPFRC are densely distributed in the pure bending section and nearby areas. Compared with the steel-UHPFRC composite plates, the composite beams are damaged by the yielding of the U-rib rather than the yielding of the reinforcement bars. In addition, the ultimate bearing capacity of composite plates remains basically unchanged after the yielding of reinforcement. On the other hand, the maximum crack width and propagation speed are significantly smaller than those of steel-UHPFRC composite plates [15], that is, the bending performance of LWCD along the transverse and longitudinal directions is quite different.

#### 4.3.2. Influence of Main Design Parameters on Cracking Load

According to studies by Rafiee [30], when the maximum crack width in UHPFRC is not larger than 0.05 mm, the cracks should not influence the durability of UHPFRC. Furthermore, those cracks with widths less than 0.05 mm are difficult to be found by naked eyes in practical engineering [31]. Therefore, the corresponding load when the maximum crack width of the UHPFRC surface reaches 0.05 mm is called the cracking load. The cracking stress of specimens can be calculated according to the conversion section method. The main test results are given in Table 4.

As can be seen from Table 4, the cracking stresses of the members are between 20 MPa to 23.5 MPa. The cracking stresses of steel-UHPFRC composite beam with 45 mm thickness of UHPFRC layer are between 20.6 MPa to 27.3 MPa, and for the components with the thickness of UHPFRC layer of 60 mm, the cracking stresses are between 20.0 MPa to 25.2 MPa. When other parameters remain unchanged and the cover thickness is reduced from 25 mm to 15 mm, the cracking stress of the steel-UHPFRC composite beam increases by 7.2–13.5%, that is, reducing the cover thickness can increase the cracking stress of components. When the thickness of the protective layer and UHPFRC layer remains unchanged and the number of longitudinal reinforcement increases from 12 to 16, the cracking stress of members increases by 6.2–20.4%. Therefore, increasing the reinforcement ratio can also improve the cracking stress of members. When the thickness of the UHPFRC layer increases from 45 mm to 60 mm, the cracking stress of components decreases by 2.9–11.6%. Therefore, increasing the thickness of the UHPFRC layer cannot increase the cracking stress.

#### 4.3.3. Analysis of Interface Slip Characteristics

**Figure 13:**
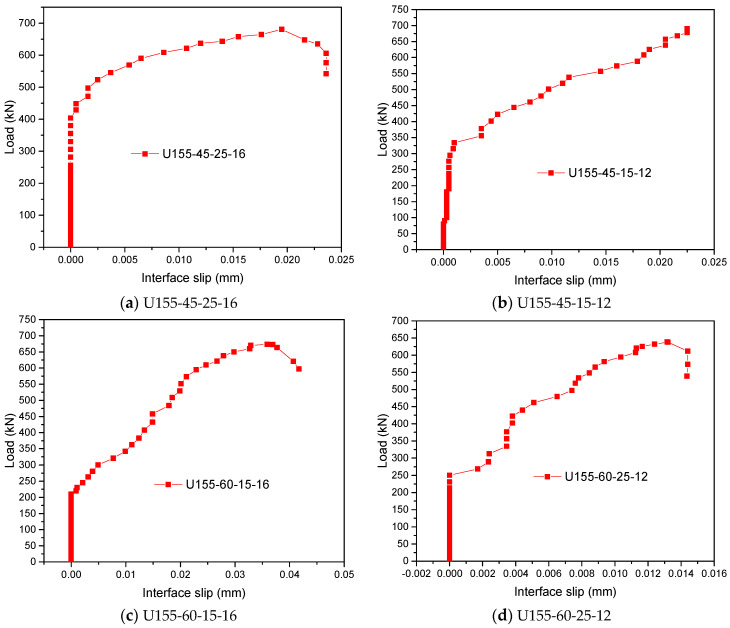
Load-interface slip curves of some members.

The slip between the steel and UHPFRC layer interface is measured by dial indicators S1 and S2 (Figure 8a) set at the end. Results take the average value of measured slip values, and draw the load-interface slip curve of some members from the beginning of loading to near the ultimate bearing capacity, as shown in Figure 13. It can be seen that the characteristics of load-interface slip curves are similar: when the load is small, there is no slip at the interface between the steel and the UHPFRC layer. When the load continues to rise, the interface begins to occur slip and gradually increases with the increase of load. Overall, the slip between the steel and UHPFRC layer of the steel-UHPFRC composite beam is small. When the load reaches the ultimate bearing capacity state, the maximum slip value of the member is about 0.013 mm–0.035 mm. The interface between the steel and UHPFRC layer is intact, and no damage is observed.

#### 4.3.4. Strain Characteristics Analysis of UHPFRC Surface

**Figure 14:**
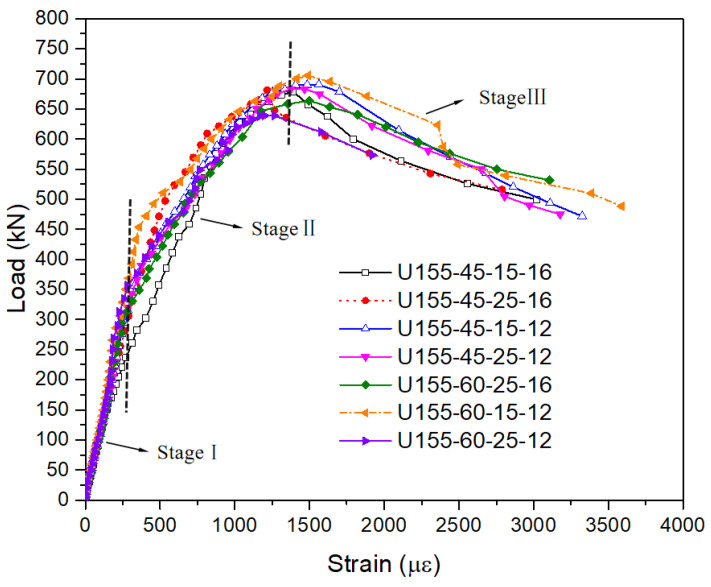
Load- strain of UHPFRC surface curves.

According to the strain data under each level of load measured in the tests, take the average value of 15 concrete strain gauges (U1-U15) on the surface of UHPFRC and draw the load-strain of the UHPFRC surface curve, as shown in Figure 14.

It can be seen from Figure 14 that the characteristics of the strain of UHPFRC surface curves are similar, which can be divided into three stages. In stage Ⅰ, the load-strain curve is approximate to a straight line, that is, the strain of the UHPFRC surface increases linearly with the load, and the curves of each component basically coincide. According to statistics, the strain of UHPFRC top surface under cracking load is between 460 με and 920 με. In stage II, the rate of UHPFRC surface strain increases with the increase of load, and there is a certain difference in the load-UHPFRC surface strain curve between components. The reason is that the key design parameters of each component are different, and the parameters such as reinforcement ratio and cover thickness have a certain impact on the strain of the UHPFRC surface. In addition, due to the heterogeneity of UHPFRC material itself, the location and expansion of cracks on the upper surface of UHPFRC have a certain randomness. Under the action of ultimate bearing capacity, the strain of the UHPFRC top surface of each specimen is between 1217 με and 1563 με. In stage III, the U-rib buckles and the load decreases, but the surface strain of UHPFRC increases rapidly.

## 5. Ultimate Bearing Capacity Calculation Theory of Composite Beam

In order to predict the ultimate bearing capacity of steel-UHPFRC composite beam, the calculation theory of ultimate bearing capacity is discussed here on the basis of the above tests.

**Figure 15:**
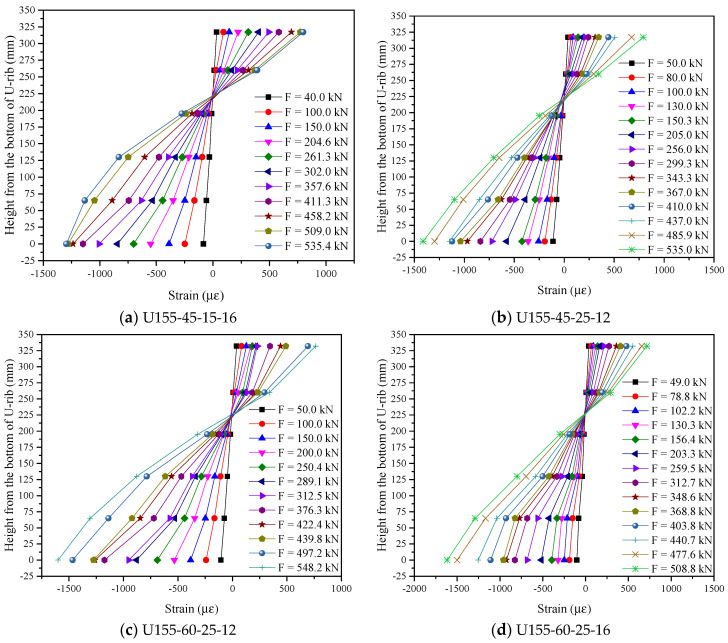
Strain distribution diagrams along the height direction.

According to the test results, when the load reached the ultimate bearing capacity state, the bottom of the U-rib in the compression area yielded, and fine cracks appeared on the UHPFRC layer surface in the tension area. Due to the bridging effect of steel fibers, the UHPFRC does not quit its work after cracking, which is different from ordinary concrete. The maximum slip value of the member was small (0.013 mm–0.035 mm) and no damage was observed at the steel-concrete interface. In addition, the strain distribution diagrams along the height direction of the member under different loads were drawn according to the actually measured strain data (take some components as an example), as shown in Figure 15. As can be seen from Figure 15, when the load is at a relatively low level (When it is lower than 76.7–86% of the ultimate load), the strain presents a linear distribution. With the increase of load, the nonlinear trend gradually appears. However, for steel-UHPFRC composite beams, the strain distribution along the height direction basically conforms to the plane section assumption.

**Figure 16:**
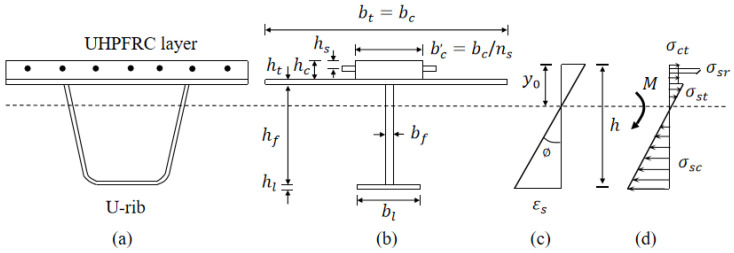
Stress diagram of ultimate bearing capacity calculation. (**a**) Original section (**b**) Equivalent section (**c**) Distribution of strain (**d**) Stress.

Based on the above reasons, in order to simplify the calculation, the following assumptions are made for the calculation of the ultimate bearing capacity of the steel-UHPFRC composite beam. (1) In the ultimate stress state, the cracking UHPFRC in the tensile zone participates in the stress, and remains unchanged after reaching the axial tensile strength; (2) The strain distribution conforms to the plane section assumption, and the strain changes linearly along the height direction; (3) The bottom of the U-rib has reached the yield state. Based on the above assumptions, the stress diagram of the ultimate bearing capacity calculation of the steel-UHPFRC composite beam is shown in Figure 16.

Under the action of ultimate bearing capacity, the internal force and moment balance equation of each section can be written as Equations (1)–(3).

(1) $$N_{sc}=N_{ct}+N_{st}+N_{sr}$$

(2) $$M_{\max}=M_{sc}+M_{ct}+M_{st}+M_{sr}$$

(3) $$F_{\max}=\frac{2M_{\max}}{L}$$

According to the strain and stress distribution of the section in Figure 16, the internal force ($N_{ct}$) and bending moment ($M_{ct}$) of UHPFRC in the tensile area can be expressed as:

(4) $$N_{ct}=f_{ct}h_{c}b_{c}$$

(5) $$M_{ct}=f_{ct}h_{c}b_{c}(y_{0}-\frac{h_{c}}{2})$$

The internal force and bending moment of the reinforcement in the tensile area ($N_{sr}$ and $M_{sr}$) can be expressed as:

(6) $$N_{sr}=\frac{A_{s}E_{s}\varepsilon_{s}(y_{0}-h_{s})}{h-y_{0}}$$

(7) $$M_{sr}=\frac{A_{s}E_{s}\varepsilon_{s}{\left(y_{0}-h_{s}\right)}^{2}}{h-y_{0}}$$

The internal force and bending moment of steel structure in tension area ($N_{st}$ and $M_{st}$) and compression area ($N_{sc}$ and $M_{sc}$) can be written as

(8) $$N_{st}=\frac{b_{t}h_{t}E_{s}\varepsilon_{s}(y_{0}-h_{c}-\frac{h_{t}}{2})}{h-y_{0}}+\frac{b_{f}E_{s}\varepsilon_{s}{\left(y_{0}-h_{c}-h_{t}\right)}^{2}}{2(h-y_{0})}$$

(9) $$M_{st}=\frac{b_{t}E_{s}\varepsilon_{s}\left[{\left(y_{0}-h_{c}\right)}^{3}-{\left(y_{0}-h_{c}-h_{t}\right)}^{3}\right]}{3(h-y_{0})}+\frac{b_{f}E_{s}\varepsilon_{s}{\left(y_{0}-h_{c}-h_{t}\right)}^{3}}{3(h-y_{0})}$$

(10) $$N_{sc}=\frac{b_{f}E_{s}\varepsilon_{s}{\left(h_{c}+h_{t}+h_{f}-y_{0}\right)}^{2}}{2(h-y_{0})}+\frac{b_{l}h_{l}E_{s}\varepsilon_{s}(h_{c}+h_{t}+h_{f}+\frac{h_{l}}{2}-y_{0})}{h-y_{0}}$$

(11) $$\begin{array}{l} M_{sc}=\frac{b_{f}E_{s}\varepsilon_{s}{\left(h_{c}+h_{t}+h_{f}-y_{0}\right)}^{3}}{3(h-y_{0})}+\\ \frac{b_{l}E_{s}\varepsilon_{s}\left[{\left(h_{c}+h_{t}+h_{f}+h_{l}-y_{0}\right)}^{3}-{\left(h_{c}+h_{t}+h_{f}-y_{0}\right)}^{3}\right]}{3(h-y_{0})} \end{array}$$

where, the meanings represented by the symbols in Equations (4)–(11) are indicated in Figure 16, $f_{ct}$ represents the axial tensile strength of UHPFRC and $E_{s}$ represents the modulus of elasticity of steel. Thus, the ultimate bearing capacity of the steel-UHPFRC composite beam can be obtained from Equations (1) to (11). It should be noted that the actual yield strength of Q345 steel measured by the material property test is too large, which is about 1.4 times the design yield strength, as shown in Figure 5b. In order to facilitate the calculation and be safe, the strain at the bottom of the U rib ($\varepsilon_{s}$) is calculated by taking the yield strain and 1.2 times the yield strain, respectively, and the calculated value of the ultimate bearing capacity of the composite beam is recorded as A and B, as shown in Table 5.

It can be seen from Table 5 that when the strain value at the bottom of the u rib is the design yield strain, compared with the test value, the calculated ultimate bearing capacity is small, and the relative error is large (about 12.2–21.3%). The reason is that the bottom of u rib has yielded under the ultimate bearing capacity state, and the actual yield strain of steel is greater than the standard yield strain, so this method is too conservative. While the calculated results of ultimate bearing capacity (calculated value B) are in good agreement with the experimental values, and the relative error is within 7%. In addition, the theoretical calculation formula is partial to safety, which can provide a reference for the design of reinforced steel-UHPFRC composite deck structure.

## 6. Fatigue Testing of the Steel-UHPFRC Composite Plate

### 6.1. Design of the Steel-UHPFRC Composite Plate

**Figure 17:**
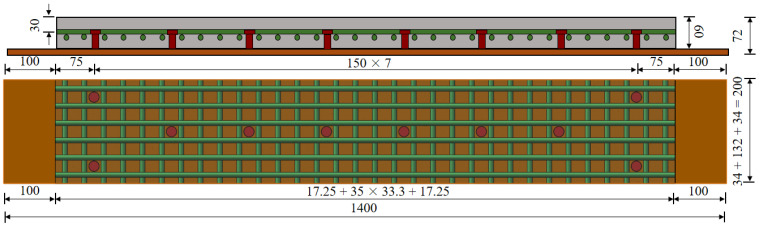
Structural diagram of specimen S150-60-25-6 (unit: mm).

In order to reveal the fatigue endurance of steel-UHPFRC composite deck structures in the transverse direction, one steel-UHPFRC composite plate with dimensions of 1200 mm (length) × 200 mm (width) × 72 mm (height) was fabricated, as shown in Figure 17. The UHPFRC layer was reinforced by steel rebars with diameters of 10 mm and the spacing in both longitudinal and transverse directions was 33.3 mm. The stud spacing was 150 mm and the cover thickness was 25 mm. The material properties of UHPFRC, reinforcement bar, and steel are the same as those in Section 3.2. The manufacturing and curing process of components is shown in Section 3.3.

### 6.2. Loading Scheme and Test Contents

**Figure 18:**
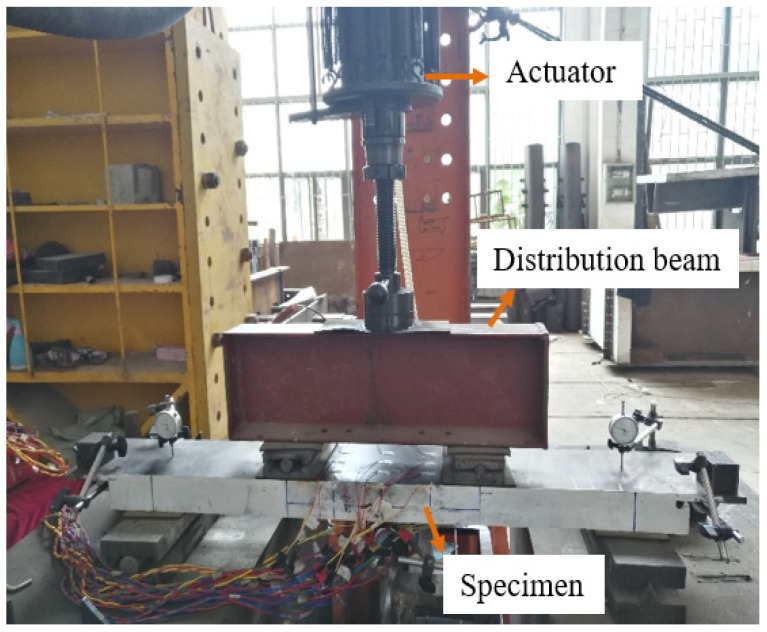
The fatigue test.

The four-point negative bending test method was used, and the loading diagram of the fatigue test is shown in Figure 18. The length of the pure negative bending zone was 400 mm and allow for convenient observation of crack formation and propagation. The load was applied to the distribution beam through a hydraulic jack. The specimen was loaded at a constant-amplitude fatigue force under design stress. The loading frequency was 4.0 Hz. The minimum fatigue load ($P_{min}$) was 10% of the maximum fatigue load ($P_{max}$). When the number of fatigue cycles exceeded 10 million, the fatigue stress amplitude was increased. The specific load and stress amplitude in UHPFRC was shown in Table 6. After a certain number of fatigue cycles, the fatigue test was stopped and a static loading test was carried out. The instrumentation and test method used to measure the crack width, mid-span displacement, and slip were the same as for the steel-UHPFRC composite plate.

### 6.3. Experimental Results Analysis of Fatigue Test

**Figure 19:**
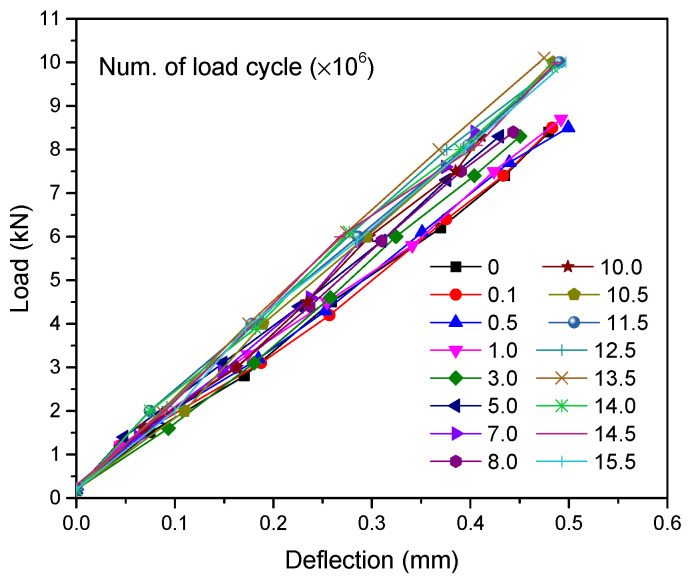
Load-span deflection curve.

After 17.6 million cycles, the crack width reached 0.1 mm, and then the fatigue test was completed. The load-deflection curve is shown in Figure 19, where only a small loss of flexural stiffness was observed based on the curve slopes. According to the Palmgren–Miner linear cumulative rule [32], different stress ranges satisfy the relationship as follows:

(12) $$N^{\prime }=\overset{n}{\underset{i=1}{\sum }}{\left(\frac{\sigma_{i}}{\sigma^{\prime }}\right)}^{m}n_{i}$$

where $\sigma^{\prime }$= design stress (in MPa); $\sigma_{i}\;$= random stress range (in MPa); m = slope of the *S*-*N* curve; the value of which is 3.0; $n_{i}$ = loading cycles for the ith random load; and $N^{\prime }$ = equivalent loading cycles of the design load.

As mentioned above in Section 2.3, the maximum tensile stress of UHPFRC under design load in the transverse bridge direction is 3.8 MPa, the design stress is 4.5 MPa conservatively; the random load refers to the load in the fatigue test is shown in Table 6, substituting these values into Equation (12), the equivalent number of load cycles can be calculated under the design fatigue stress amplitude, as shown in Table 6.

**Figure 20:**
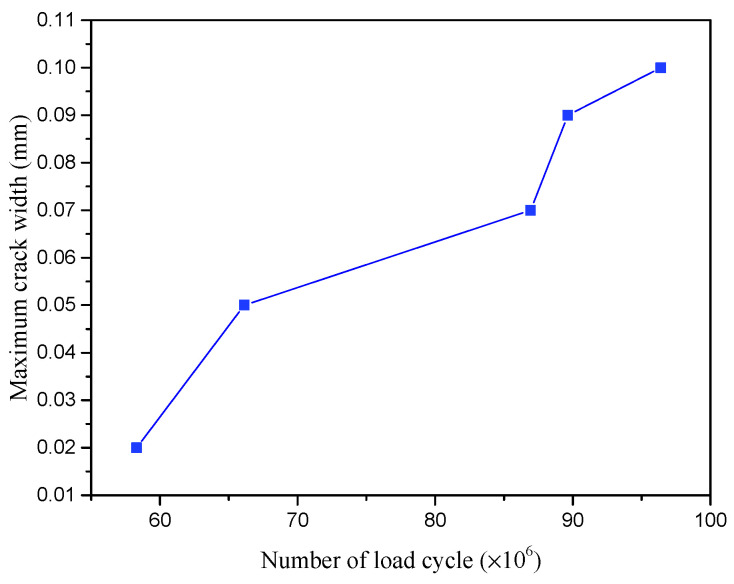
Maximum crack width- equivalent number of load cycle curve.

**Figure 21:**
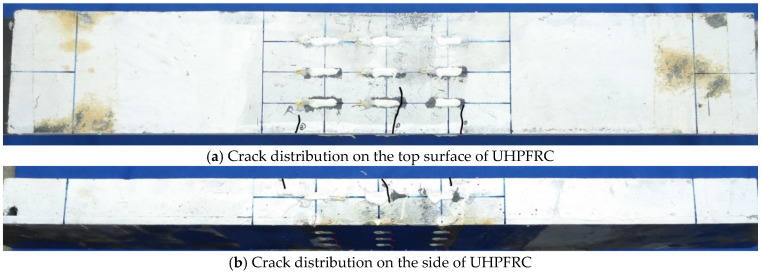
Crack distribution of specimen.

The maximum crack width versus the equivalent number of load cycle curves is shown in Figure 20. It is observed that the crack with a width of 0.05 mm appeared on the surface of UHPFRC only after 66.12 million loading cycles, which far exceeds the infinite fatigue life requirements of 10 million fatigue cycles. The design principle for the LWCD is that the maximum crack opening at the top of UHPFRC should not exceed 0.05 mm under the design load in the normal use limit state. This means that the components have good fatigue resistance in normal use limit state, meeting the design requirements. With the increase in fatigue cycles, the crack width and length increased gradually. At the end of fatigue testing after 96.4 million loading cycles, the maximum crack width reached 0.1 mm. There were three cracks on the surface of UHPFRC, as shown in Figure 21. The maximum crack length was 80 mm and the width was 0.1 mm. The other two cracks had lengths of 40 mm and 50 mm respectively, and the maximum width was 0.02 mm. In addition, the bonding surface between the steel plate and the UHPFRC layer was almost intact with only 0.03 mm of slippage.

As can be seen from the above research, the LWCD with 60-mm UHPFRC layer and 25-mm cover thickness has excellent flexural and fatigue performance and can meet the engineering design requirements.

## 7. Conclusions

This paper studies the influence of main design parameters on longitudinal bending performance and the transverse bending fatigue behavior of LWCD via FEA and full-scale model tests. Based on the tests, the calculation theory of ultimate bearing capacity of steel-UHPFRC composite beams considering UHPFRC mechanical properties is proposed, the main conclusions are as follows:

(1) FEA revealed that the maximum tensile stress of UHPFRC in the transverse bridge direction is 3.8 MPa. Adding a 60-mm UHPFRC layer can significantly reduce the stress amplitude of six typical fatigue details and the reduction degree is 44.8% to 90%. Accordingly, the risk of fatigue cracking can be greatly reduced.
(2) For the longitudinal bending performance of the LWCD, the steel-UHPFRC composite beams would fail due to the buckle of the steel U-rib. The cracking stresses of the specimens are between 20.0 MPa to 27.3 MPa. Reducing the cover thickness and increasing the reinforcement ratio can effectively improve the cracking stress of specimens. However, increasing the thickness of the UHPFRC layer cannot increase the cracking stress. The ultimate bearing capacity of specimens with different parameters has little difference.
(3) The load-deflection curves of steel-UHPFRC composite beams include three different stages, namely, the elastic stage, crack expansion stage, and buckle stage. All specimens exhibit multiple cracking behaviors when components failed. The maximum slip value between the steel and UHPFRC layer is about 0.013 mm–0.035 mm and no damage is observed. In addition, the strain distribution along the height direction basically conforms to the plane section assumption.
(4) According to the test results, considering the force of cracking UHPFRC in the tensile zone, the calculation method of the ultimate bearing capacity of the steel-UHPFRC composite structure is proposed. When the strain at the bottom of the u-rib is taken as 1.2 times the design yield strain, the calculated results are in good agreement with the experimental results.
(5) The transverse fatigue test results revealed that the specimen can experience 66.12 million loading cycles under the design fatigue stress amplitude. In addition, only a little loss of stiffness was observed, and the value of slip between steel plate and UHPFRC layer was small. This indicates that the LWCD has good fatigue resistance in the transverse direction.

## Figures and Tables

**Table 1:** Stress amplitude calculation results in typical fatigue details.

Details		Detail ①	Detail ②	Detail ③	Detail ④	Detail ⑤	Detail ⑥
Maximum stress amplitude (MPa)	0-mm UHPFRC	84.79	61.02	90.37	120.56	79.61	67.15
	60-mm UHPFRC	8.28	23.99	38.9	58.45	43.91	35.21
Reduction degree in stress amplitude		90%	60.7%	57%	51.5%	44.8%	47.6%

**Table 2:** Summary of test program.

Serial No.	Specimen Name	Stud Spacing (mm)	UHPFRC Thickness (mm)	Cover Thickness (mm)	Number of Steel Bars	Reinforcement Ratio	Test Type
1	U155-45-15-12	155	45	15	12	3.4%	Longitudinal bending test
2	U155-45-15-16	155	45	15	16	4.5%	
3	U155-45-25-12	155	45	25	12	3.4%	
4	U155-45-25-16	155	45	25	16	4.5%	
5	U155-60-15-12	155	60	15	12	2.5%	
6	U155-60-15-16	155	60	15	16	3.4%	
7	U155-60-25-12	155	60	25	12	2.5%	
8	U155-60-25-16	155	60	25	16	3.4%	
9	S150-60-25-6	150	60	25	6	3.9%	Fatigue test

Notes: S denotes the specimen of the steel-UHPFRC composite plate, U denotes the specimen of the steel-UHPFRC composite beam, 150/155 represents the stud spacing, 60 represents the thickness of UHPFRC is 60 mm, 25 represents the cover thickness is 25 mm, 6/12/16 specify the number of Φ10-mm reinforcement bars along the stress direction.

**Table 3:** Mechanical properties of UHPFRC.

Serial No.	Test Type	Dimensions of Specimen (mm)	Number of Specimen	Fiber Parameter	Mechanical Property	Test Result
1	compression test	100 × 100 × 100	3	1.5% straight fiber (Φ 0.12 × 8 mm) + 2% end-hook fiber (Φ 0.2 × 13 mm)	compressive strength	165.0 MPa
2	elastic modulus test	100 × 100 × 300	6		modulus of elasticity	45.8 GPa
3	flexural test	100 × 100 × 400	3		flexural strength	30.1 MPa

**Table 4:** Main test results of steel-UHPFRC composite beams.

Specimen	Reinforcement Ratio	Cover Thickness	Cracking Load (kN)	Cracking Stress (MPa)
U155-45-15-16	4.5%	15	593	27.3
U155-45-25-16	4.5%	25	523.3	24.8
U155-45-15-12	3.4%	15	538.4	25.7
U155-45-25-12	3.4%	25	437	20.6
U155-60-15-16	3.4%	15	551.2	25.2
U155-60-25-16	3.4%	25	508.8	23.5
U155-60-15-12	2.5%	15	492.5	22.7
U155-60-25-12	2.5%	25	439.8	20.0

**Table 5:** Calculated value and comparison of the ultimate bearing capacity.

Specimen	Calculated Value A/kN	Calculated Value B/kN	Test Value C/kN	(C-A)/C	(C-B)/C
U155-45-15-16	545.9	645.6	678.6	19.6%	4.9%
U155-45-25-16	541.4	640.1	680.9	20.5%	6.0%
U155-45-15-12	543.3	642.2	690.3	21.3%	7.0%
U155-45-25-12	539.7	637.8	683.0	21.0%	6.6%
U155-60-15-16	569.3	670.3	673.5	15.5%	0.5%
U155-60-25-16	563.9	663.7	663.7	15.0%	0.0%
U155-60-15-12	565.1	664.9	705.4	19.9%	5.7%
U155-60-25-12	560.8	659.6	639.0	12.2%	−3.2%

**Table 6:** Specific load and stress amplitude of fatigue test.

$P_{max}(\mathrm{kN})$	$P_{min}(\mathrm{kN})$	Stress Amplitude (MPa)	$\mathrm{Number}\;\mathrm{of}\;\mathrm{Load}\;\mathrm{Cycles}(\times 10^{6})$	$\mathrm{Cumulative}\;\mathrm{Equivalent}\;\mathrm{Number}\;\mathrm{of}\;\mathrm{Load}\;\mathrm{Cycles}(\times 10^{6})$
8.4	0.8	4.5	10.0	10.0
15.1	1.5	8.1	3.5	30.4
18.4	1.8	9.9	2.0	51.7
21.7	2.2	11.7	1.3	74.1
25.0	2.5	13.5	0.8	96.4

## References

[1] Pfeil M.S., Battista R.C., Mergulhão A.J. (2005). Stress concentration in steel bridge orthotropic decks. J. Constr. Steel Res..

[2] Wolchuk R. (2014). Empirical Design Rules for Effective Utilization of Orthotropic Decks. J. Bridg. Eng..

[3] Shao X., Yi D., Huang Z., Zhao H., Chen B., Liu M. (2013). Basic Performance of the Composite Deck System Composed of Orthotropic Steel Deck and Ultrathin RPC Layer. J. Bridg. Eng..

[4] Shen X.J., Brühwiler E., Peng W.H. (2020). Biaxial flflexural response of Strain-Hardening UHPFRC circular slab elements. Int. J. Fatigue.

[5] Shen X.J., Brühwiler E. (2020). Biaxial flexural fatigue behavior of strain-hardening UHPFRC thin slab elements. Constr. Build. Mater..

[6] Noshiravani T., Brühwiler E. (2013). Experimental investigation on reinforced ultra-high-performance fiber-reinforced concrete composite beams subjected to combined bending and shear. Aci Struct. J..

[7] Prem P.R., Murthy A.R. (2016). Acoustic emission and flexural behaviour of RC beams strengthened with UHPC overlay. Constr. Build. Mater..

[8] Tanarslan H.M., Alver N., Jahangiri R., Yalçınkaya Ç., Yazıcı H. (2017). Flexural strengthening of RC beams using UHPFRC laminates: Bonding techniques and rebar addition. Constr. Build. Mater..

[9] Lorenc W., Kubica E. (2006). Behavior of composite beams prestressed with external tendons: Experimental study. J. Constr. Steel Res..

[10] Buitelaar P., Braam R., Kaptijn N. Reinforced high performance concrete overlay system for rehabilitation and strengthening of orthotropic steel bridge decks. Proceedings of the ASCE/SEI Orthotropic Bridge Conference.

[11] Dieng L., Marchand P., Gomes F., Tessier C., Toutlemonde F. (2013). Use of UHPFRC overlay to reduce stresses in orthotropic steel decks. J. Constr. Steel Res..

[12] Choi W., Choi Y.-C., Yoo S.-W. (2018). Flexural Design and Analysis of Composite Beams with Inverted-T Steel Girder with Ultrahigh Performance Concrete Slab. Adv. Civ. Eng..

[13] Li W.G. (2015). Experimental Research on Static and Fatigue Flexural Performance of UHPC Layer in Light-Weighted Composite bridge Deck. Master’s Thesis.

[14] Shao X.D., Qu W.T., Cao J.H., Yao Y.L. (2018). Static and fatigue properties of the steel-UHPC lightweight compositebridge deck with large U-ribs. J. Constr. Steel. Res..

[15] Luo J., Shao X.D., Cao J.H., Xiong M.H., Fan W. (2019). Transverse bending behavior of the steel-UHPC lightweight composite deck: Orthogonal test and analysis. J. Constr. Steel Res..

[16] Zhang S., Shao X., Cao J., Cui J., Hu J., Deng L. (2016). Fatigue Performance of a Lightweight Composite Bridge Deck with Open Ribs. J. Bridg. Eng..

[17] Ding N., Shao X.D. (2015). Study on fatigue performance of light-weighted composite bridge deck. China Civ. Eng. J..

[18] Shao X., Cao J. (2018). Fatigue Assessment of Steel-UHPC Lightweight Composite Deck Based on Multiscale FE Analysis: Case Study. J. Bridg. Eng..

[19] Pei B., Li L., Shao X., Wang L., Zeng Y. (2018). Field measurement and practical design of a lightweight composite bridge deck. J. Constr. Steel Res..

[20] Kożuch M., Lorenc W. (2018). Stress concentration factors of shear connection by composite dowels with MCL shape. Arch. Civ. Mech. Eng..

[21] Lorenc W. (2016). The design concept for the steel part of a composite dowel shear connection. Steel Constr..

[22] Lorenc W. (2017). The model for a general composite section resulting from the introduction of composite dowels. Steel Constr..

[23] Shao X.D., Hu J.H. (2015). The Steel-UHPC Lightweight Composite Bridge Structures.

[24] Zhan J., Shao X.D., Qu W.T., Cao J.H. (2018). Multi-parametric Analysis on steel-STC lightweight composite bridge deck. J. Highw. Transp. Res. Dev..

[25] Ministry of Communications of China (2015). Specifications for Design of Highway Steel Bridge (JTG D64-2015).

[26] Sim H.B., Uang C.M., Sikorsky C. (2009). Effects of Fabrication Procedures on Fatigue Resistance of Welded Joints in Steel Orthotropic Decks. J. Bridge Eng..

[27] Wolchuk R. (1963). Design Manual for Orthotropic Steel Plate Deck Bridges.

[28] (2015). Reactive Powder Concrete.

[29] AFGC/SETRA (2016). Ultra High Performance Fibre-Reinforced Concretes.

[30] Rafiee A. (2012). Computer Modeling and Investgation on the Steel Corrosion in Cracked Ultra High Performance Concrete.

[31] Makita T., Brühwiler E. (2013). Tensile fatigue behaviour of ultra-high performance fibre reinforced concrete (UHPFRC). Mater. Struct..

[32] AASHTO (2012). AASHTO LRFD Bridge Design Specification.

